# A Bluetooth/PDR Integration Algorithm for an Indoor Positioning System

**DOI:** 10.3390/s151024862

**Published:** 2015-09-25

**Authors:** Xin Li, Jian Wang, Chunyan Liu

**Affiliations:** 1 School of Computer Science and Technology, China University of Mining and Technology, Xuzhou 221116, China; 2 School of Environmental Science and Spatial Informatics, China University of Mining and Technology, Xuzhou 221116, China; E-Mails: wjian@cumt.edu.cn (J.W.); xzlcy2015@126.com (C.L.)

**Keywords:** bluetooth beacons, pedestrian dead reckoning, adaptive system noise, extended kalman filter, map matching

## Abstract

This paper proposes two schemes for indoor positioning by fusing Bluetooth beacons and a pedestrian dead reckoning (PDR) technique to provide meter-level positioning without additional infrastructure. As to the PDR approach, a more effective multi-threshold step detection algorithm is used to improve the positioning accuracy. According to pedestrians’ different walking patterns such as walking or running, this paper makes a comparative analysis of multiple step length calculation models to determine a linear computation model and the relevant parameters. In consideration of the deviation between the real heading and the value of the orientation sensor, a heading estimation method with real-time compensation is proposed, which is based on a Kalman filter with map geometry information. The corrected heading can inhibit the positioning error accumulation and improve the positioning accuracy of PDR. Moreover, this paper has implemented two positioning approaches integrated with Bluetooth and PDR. One is the PDR-based positioning method based on map matching and position correction through Bluetooth. There will not be too much calculation work or too high maintenance costs using this method. The other method is a fusion calculation method based on the pedestrians’ moving status (direct movement or making a turn) to determine adaptively the noise parameters in an Extended Kalman Filter (EKF) system. This method has worked very well in the elimination of various phenomena, including the “go and back” phenomenon caused by the instability of the Bluetooth-based positioning system and the “cross-wall” phenomenon due to the accumulative errors caused by the PDR algorithm. Experiments performed on the fourth floor of the School of Environmental Science and Spatial Informatics (SESSI) building in the China University of Mining and Technology (CUMT) campus showed that the proposed scheme can reliably achieve a 2-meter precision.

## 1. Introduction

Location Based Services (LBS) are mobile applications which rely on a user’s location to deliver context aware functionality. Industry forecasts for this area predict huge market growth and revenue. The Global Positioning System (GPS) is the most popular positioning system; however, it is not suitable for indoor positioning [1,2,3]. Real-time indoor positioning is still a challenge using existing techniques [4].

There are multiple technologies for indoor positioning, e.g., WiFi, ZigBee, Bluetooth, inertial navigation systems (INSs), and laser scanning systems (LSSs). Existing technologies for wireless indoor location systems such as WiFi or ZigBee system are reviewed by Pahlavan and Li [5,6,7,8]. A self-contained sensors, such as gyroscopes, accelerometers or magnetometers are used for another kind of indoor location system [4,9,10,11]. Developing a hybrid scheme for real-time indoor navigation is a more effective practice [12,13,14,15]. Afzal *et al.* used a relatively stable data aided inertial navigation device for the gravity field and geomagnetic field to regard the heading error as the estimated quantity, as well as used EKF to achieve data fusion, thereby gaining reliable heading data [16]. Wang *et al.* proposed the algorithm of dividing the region, and used a particle filter and map matching method, thereby gaining the navigation results with meter-level error [17]. Aicardi *et al.* integrated the data captured from mobile phone camera into indoor pedestrian dead reckoning, and used image matching to achieve positioning [18,19]. Gusenbauer *et al.* conducted machine learning for the data captured, as well as conducting analysis of human movement, thereby obtaining the moving distance and direction; the cumulated error of final position after moving 233 m was only 2.76% [20]. On the one hand, most of the existing methods may need additional information such as image and magnetic field, which can not only increase the volume and power consumption of the system, but also be more easily influenced by the external environment. On the other hand, most of the existing methods need large data calculation, which is suitable for post processing analysis; furthermore, it requires high operational capability of the processor, which is not suitable for application of a low cost processor. This paper focuses on researching the integration of PDR and Bluetooth with better practicability, since a Bluetooth Beacon that can be deployed easily is able to work immediately as long as it is powered by batteries.

Step detection, step length estimation and heading determination are involved in PDR algorithms [4,21]. Three types of step detection algorithms include peak detection, flat-zone detection and zero-crossing detection. If the thresholds are not appropriately set, the deficiencies of the peak and zero-crossing detection algorithms will create the potential for missing detection; or over-detection may occur in the case of the flat-zone detection algorithm as the flat-zone test statistics vary with differences in walking patterns [22]. There are considerable numbers of studies for improving the accurate estimation of step length. Techniques that have been developed for this purpose are mainly constant/quasi-constant models, linear models, nonlinear models, as well as artificial intelligence models [23]. As for a look-up table, a few levels of step length are conveniently stored for a given pedestrian on the basis of his/her locomotion mode and time duration of each step [24]. Step length can be estimated by the linear relationship between step length and frequency. With utilization of the correlation between vertical acceleration and walking velocity, Kourogi and Kurata computed the walking speed and estimated the step length through multiplying the walking speed by the time of the unit cycle of locomotion [21]. A neural network for step length estimation is presented by Cho, which is unaffected by accelerometer bias and gravity acceleration [25]. Both gyroscope and magnetometer are two types of heading sensors, which are typically used when the PDR algorithm is applied [26]. Xiao and Klingbeil proposed the concept of magnetic azimuth correcting based on gyro data collected over a short time; therefore, the heading angles are allowed to be estimated through a combination of gyroscope and magnetometer measurements [26,27]. A biaxial magnetic compass can possibly be used for calculating the azimuth after compensating for compass inclination using a shoe-mounted accelerometer [25]. An Inertial Navigation System/Extended Kalman Filter (INS/EKF) framework used to reduce heading drift has been demonstrated [11]. A detector has been proposed to perform magnetic field measurements, which can be used for accurate heading estimation. This detector uses different magnetic field test parameters which can be analyzed for good magnetic field measurements, and the mean error is controlled within 9 degrees or so [28]. There is one factor limiting the use of PDR alone for indoor navigation. It is susceptible to cumulative errors over time. 

The Bluetooth Beacon-based positioning technology is a brand-new positioning technology [29] proposed in recent years. Through the application of Bluetooth technology with low power consumption, a signal zone will be created automatically in a Beacon base station. Then, when the devices are brought into this area, their Bluetooth signals will be sensed by the relevant application program to serve for such applications as positioning or information forwarding, *etc.* Since the intensity of Beacon signal is indicated with a Received Signal Strength Indication (RSSI) value, then it is feasible to evaluate the distance between the user and the Beacon device according to the changes in the RSSI values. Actually, the Bluetooth-based positioning system has the absolute advantage in the positioning since real position coordinates have been written in it. However, due to the stochastic instability of the signal, chattering might occur in the positioning result.

To overcome these constraints, this paper constitutes a study related to fusion positioning through Bluetooth and PDR methods with the details provided below:

(1) Aiming at the indoor dead-reckoning positioning approach based on inertial technology, this paper proposes a peak-valley detection algorithm for multi-threshold step detection to identify the pedestrian gait. Through the contrast and analysis of the multiple models regarding the step length calculations in the walking mode and the running mode, this paper identifies the optimal step length calculation method that is able to satisfy simultaneously the requirement for different movement modes in addition to the provision of the relevant parameters. Also, through the research and analysis of the heading correction method, this paper proposes a heading estimation method with real-time compensation based on a Kalman filter according to the map geometry information to restrain the error accumulation, increasing the accuracy of heading calculation and improving the positioning accuracy of PDR algorithm. 

(2) Through the research and analysis of the positioning technology integrated with Bluetooth and PDR, this paper proposes two fusion models based on different principles separately through the following two methods: one has been integrated with PDR algorithm and the position correction through Bluetooth according to map matching and the other method is the adaptive noise extended Kalman filter method integrated with PDR and Bluetooth. Through the former approach, there will not be too much calculation work with low costs incurred in the deployment and maintenance, as the Kalman filtering algorithm and numerous Beacons will not be needed. However, when the latter approach is adopted, the deployment and maintenance will be costly. However, it will provide higher positioning accuracy, and the mean positioning error can be controlled within 2 m or so.

The remainder of the paper is organized as follows: In Section 2, we conduct a brief analysis of the Bluetooth-based point positioning technology, and in Section 3, propose an improved PDR algorithm. Subsequently, two Bluetooth/PDR integration schemes based on Bluetooth-based position correction and adaptive system noise EKF are demonstrated in Section 4. Finally, several experiments are analyzed in Section 5, and Section 6 concludes the paper.

## 2. Beacon-Based Point Positioning

Here are three values that describe the power of a Beacon’s signal: Broadcasting Power, RSSI and Measured Power. Broadcasting Power is the power with which the Beacon broadcasts its signal, *i.e.*, the power with which the signal leaves the Beacon’s antenna. The owner of the Beacon can change this setting. The value ranges between −23 dBm and +4 dBm, the lowest to the highest power settings, respectively. The higher the power, the bigger the Beacon’s range and the more stable the signal, but it also shortens the battery life of the Beacon. RSSI is the strength of the Beacon’s signal as seen on the receiving device, e.g., a smart phone. In general, the greater the distance between the device and the Beacon is, the lesser the strength of the received signal. This inverse relation between the distance and RSSI is used to estimate the approximate distance between the device and the Beacon using another value defined by the Beacon standard: Measured Power. Measured Power is a calibrated value which indicates the expected RSSI at a distance of one meter to the Beacon, called txPower. Combined with RSSI, this allows estimating the actual distance between the device and the Beacon. For example, we can measure a bunch of RSSI measurements at known distances, do a best fit curve to match the data points and convert the best fit curve into an algorithm.

In an ideal environment, this method is able to guarantee accurate positioning. However, since RSSI has been affected by multiple factors including signal reflection, scattering and diffraction, large errors will arise in practice. Therefore, it will be very hard to secure a 100% accurate distance measurement based on this principle. Meanwhile, when we are using Bluetooth for the positioning, we just want to define an approximate position range. Therefore, this paper does not use the distance measuring model. Instead, this paper used a pseudo threshold value of “1 m” as the txPower to detect the Bluetooth signals within 5 m.

**Figure 1:**
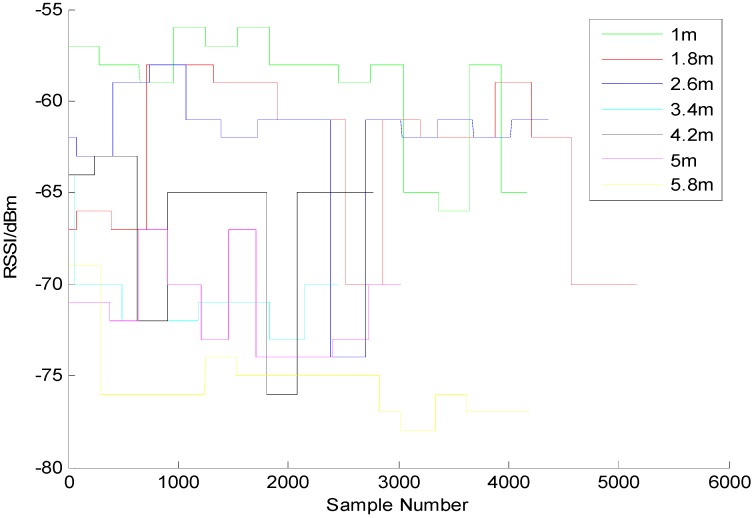
Changes in the field intensity of the beacon every 0.8 m.

**Figure 2:**
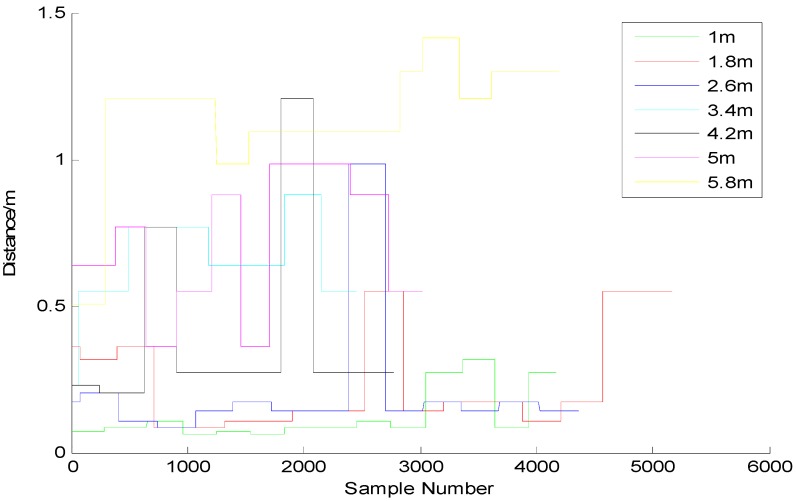
Changes in the pseudo-distance between the beacons every 0.8 m.

We turned the power up to 100% (+4 dBm), then measured the RSSI and the accuracy at the different varying distances. As shown in Figure 1, we made an observation for 2 min every other meter and recorded the changes in signal intensity to generate an intensity-distance change chart. The figure reveals that the further the distance is, the weaker the intensity is. Since we want the Beacon to have a sensing range of around 5 m, then it is necessary to set an appropriate distance as the sensing threshold for Beacon. In this case, we have set the value of txPower to be −74 dBm, which means that this value will be used as a judging threshold of “1 m”. Figure 2 shows the distance measuring result after this threshold has been applied. The observation of the changes in signal intensity and distance every 0.8 m reveals that basically all of the signal intensities within 5 m have been covered in the sensing range, indicating the application of this threshold has contributed to obtaining a sensing area with a radius of 5 m.

## 3. PDR Algorithm Based on the Inertial Sensor in Mobile Phones 

According to the PDR algorithm, the accelerometer, the magnetic sensor and the gyroscope embedded in a smart phone will be combined into an inertial measurement unit to locate the position of the mobile terminal based on the following formula:

(1) $$\left\{ \begin{array}{l} X_{i+1}=X_{i}+SL_{i}\times \sin\alpha_{i}\\ Y_{i+1}=Y_{i}+SL_{i}\times \cos\alpha_{i} \end{array}\right.$$

where (X,Y) indicates the coordinates of the position, SL is the step length and α represents the heading angle, thus the PDR algorithm mainly consists of the following three steps including gait detection, step length evaluation and the determination of heading angle.

### 3.1. Multi-Threshold Step Detection

In order to make real-time gait detection through data, this paper proposes a peak-valley detection based multi-threshold step detection model, where the following two groups of constraint conditions $\left(a_{p},\Delta a_{p},\Delta t_{p},\Delta t_{pv}\right)$and $\left(a_{v},\Delta a_{v},\Delta t_{v},\Delta t_{vp}\right)$ will be used to define the peak-valley detection constraints with:

(1) $a_{p}$ and $a_{v}$ representing separately the amplitude at the extreme point of the peak and valley on the waveform.
(2) $\Delta a_{p}$ and $\Delta a_{v}$ representing separately the amplitude difference between the adjacent peaks and between the adjacent valleys.
(3) $\Delta t_{p}$ and $\Delta t_{v}$ representing separately the time difference between two adjacent peaks and between two adjacent valleys.
(4) $\Delta t_{pv}$ and $\Delta t_{vp}$ representing separately the time difference between the adjacent peak and valley or between the adjacent valley and peak.

**Figure 3:**
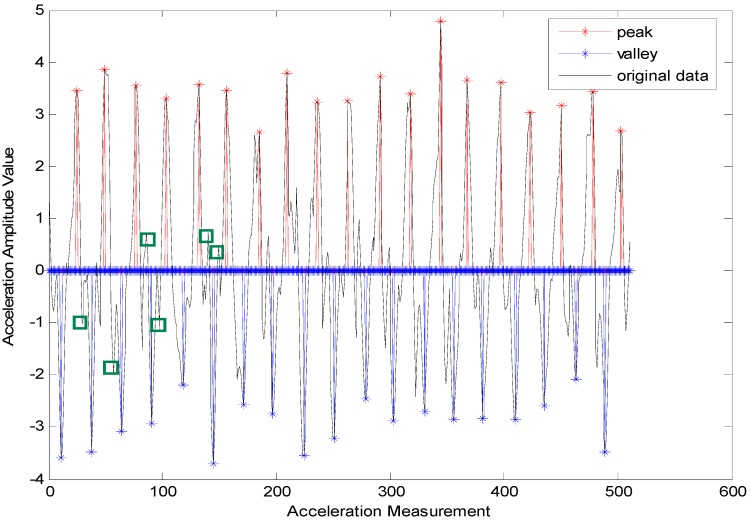
Faulty step detection induced by a pseudo-peak and a pseudo-valley.

Judge the pedestrian’s moving state, at rest or moving according to $a_{p}$ and $a_{v}$, both of which are the acceleration amplitude at the extreme point of the waveform. If it is in a static state, it indicates the ending of gait recognition. If not, it would become necessary to make a further verification of whether this value is the true value (peak/valley) in a gait cycle. Take peak detection as an instance. Set a dual time threshold $\left(\Delta t_{p},\Delta t_{pv}\right)$according to the periodicity of a complete gait cycle. In normal cases, it is applicable to set $\Delta t_{pv}=\frac{1}{2}\Delta t_{p}$. Therefore, the peak detection model can be expressed as:

(2) $$peak=\left\{ \begin{array}{l} 1，a_{p}\geq \delta_{a_{p}}\&\Delta t_{p}\geq \delta_{\Delta t_{p}}\&\Delta t_{pv}\geq \delta_{\Delta t_{pv}}\\ 0，a_{p}<\delta_{a_{p}}||\Delta t_{p}<\delta_{\Delta t_{p}}||\Delta t_{pv}<\delta_{\Delta t_{pv}} \end{array}\right.$$

where peak=1 means that the acceleration data is also the peak, peak=0 indicates that it is not a peak and $\left(\delta_{a_{p}},\delta_{\Delta t_{p}},\delta_{\Delta t_{pv}}\right)$ represents the threshold set for a peak detection. Generally, they are the empirical values. According to the experimental tests, we have $\delta_{\Delta t_{pv}}=\frac{1}{2}\delta_{\Delta t_{p}}$. If two or more continuous peaks are compliant with the above conditions (as shown in Figure 3) and are detected without the occurrence of valleys that do not conform to the constraint model, then utilize $\Delta a_{p}$, the amplitude difference between the adjacent two peaks to restrict and control the real peak according to the peak-valley synchronization criterion in a normal gait cycle and the general knowledge of peak coming first. That is to say, the following constraint condition, $\Delta a_{p}\geq 0$ must be added subsequently. Then, the real peak detection model can be expressed as below:

(3) $$peak=\left\{ \begin{array}{l} 1，a_{p}\geq \delta_{a_{p}}\&\Delta t_{p}\geq \delta_{\Delta t_{p}}\&\Delta t_{pv}\geq \delta_{\Delta t_{pv}}=\frac{1}{2}\delta_{\Delta t_{p}}\&\Delta a_{p}\geq 0\\ 0，a_{p}<\delta_{a_{p}}||\Delta t_{p}<\delta_{\Delta t_{p}}||\Delta t_{pv}<\delta_{\Delta t_{pv}}=\frac{1}{2}\delta_{\Delta t_{p}}||\Delta a_{p}<0 \end{array}\right.$$

After that, utilize the condition set $\left(a_{v},\Delta a_{v},\Delta t_{v},\Delta t_{vp}\right)$ to detect the valleys. The judgment on valleys will not be made except for the case that *Num_vy_* − *Num_pk_* = 0, which means that the number of the peaks is the same as that of the valleys according to the peak-valley synchronization criterion.

(4) $$valley=\left\{ \begin{array}{l} 1，\delta_{\Delta t_{vp}}=\frac{1}{2}\delta_{\Delta t_{v}},\;a_{v}\geq \delta_{a_{v}}\&\Delta t_{v}\geq \delta_{\Delta t_{v}}\&\Delta t_{vp}\geq \delta_{\Delta t_{vp}}\\ 0，\delta_{\Delta t_{vp}}=\frac{1}{2}\delta_{\Delta t_{v}},\;a_{v}<\delta_{a_{v}}||\Delta t_{v}<\delta_{\Delta t_{v}}||\Delta t_{vp}<\delta_{\Delta t_{vp}} \end{array}\right.$$

where valley=1 means that the acceleration data is also the valley, valley=0 indicates that it is not a valley, *Num_vy_* represents the number of the detections made on the valley and *Num_pk_* is the number of the detections made on the peaks. Similarly, if two or more continuous valleys are compliant with the above conditions detected, which also means that it complies with the condition of *Num_vy_* − *Num_pk_* = 1, then utilize the amplitude difference between two adjacent valleys, $\Delta a_{v}<0$ to determine the real valley. Hence, the multi-threshold valley detection model can be expressed as below:

(5) $$valley=\left\{ \begin{array}{l} 1，\delta_{\Delta t_{vp}}=\frac{1}{2}\delta_{\Delta t_{v}},a_{v}\geq \delta_{a_{v}}\&\Delta t_{v}\geq \delta_{\Delta t_{v}}\&(\Delta t_{vp}\geq \delta_{\Delta t_{vp}}\&\Delta t_{vp}\leq 3\delta_{\Delta t_{v}})\&\Delta a_{v}<0\\ 0，\delta_{\Delta t_{vp}}=\frac{1}{2}\delta_{\Delta t_{v}},a_{v}<\delta_{a_{v}}||\Delta t_{v}<\delta_{\Delta t_{v}}||\Delta t_{vp}<\delta_{\Delta t_{vp}}||\Delta a_{v}\geq 0 \end{array}\right.$$

In the formula above, the user’s walking mechanism has been taken into account. $\Delta t_{vp}\leq 3\delta_{\Delta t_{v}}$ indicates that the time difference between the current valley and the previous peak will not be greater than three times as much as the time threshold. $\left(\delta_{a_{v}},\delta_{\Delta t_{v}},\delta_{\Delta t_{vp}}\right)$ is the threshold set for valley detection, then we can get the following relationship, say $\left|\delta_{a_{v}}|=|\delta_{a_{p}}\right|$ and $\delta_{\Delta t_{v}}=\delta_{\Delta t_{p}}$.

In conclusion, if this multi-threshold peak-valley gait detection method is adopted, only two parameters are needed, which separately are $\delta_{a}$, the amplitude threshold and $\delta_{\Delta t}$, the time threshold for gaits. When the device is in a static condition or is close to being in a static condition, the acceleration extremes will not be greater than 0.3 m/s^2^. When the device moves with people, the acceleration extremes will vary with the movement of people. Hence it is applicable to set the parameter $\delta_{a}$ to be 1 m/s^2^ to differentiate the static state of a person from the moving status. As to$\delta_{\Delta t}$, the time threshold for gaits, an experiment is conducted as below. Collect two groups of data when people are walking normally under the premise that the devices are held horizontally: the first group of data is about people walking 56 steps (walk1) in a straight line normally and the second group of data is about people turning a corner in 122 steps (walk2). Also, collect two groups of data about running: the first group is about people trotting forward in a straight line in 45 steps (run1) and the second group is about people running to turn a corner in 86 steps (run2). The test results for when time threshold parameters are set for different gaits are outlined in Table 1.

**Table 1:** Gait recognition based on simple parameters’ model.

	Within 5%	Absolutely Accurate	Greater than 5%
walk1	0.04~0.12	0.13~0.36	<0.04 or >0.36
walk2	0.03~0.10	0.11~0.28	<0.03 or >0.28
run1	0.03~0.13 or 0.16~0.24	0.14~0.15	<0.03 or >0.24
run2	0.13~0.15	0.14	<0.13 or >0.15

Table 1 reveals that it is not feasible to set an extremely low (data in blue) or extremely high (data in red) time threshold for gaits, otherwise it will lead to a big error in detection. Especially when the threshold is set to be 0 or 0.01, no pedestrian gaits will be detected at all. The reason is that in Equation (5), the user’s walking mechanism has been taken into account, then the setting of the constraint condition, $\Delta t_{vp}\leq 3\delta_{\Delta t_{v}}$ indicates that the time difference between the current valley and the previous peak will not be greater than three times as much as the time threshold. Hence, when an extremely low time threshold is set, it would be impossible to detect the reasonable valleys, which might lead to a big error in detection. Moreover, since constraint conditions (such as $\Delta t_{v}\geq \delta_{\Delta t_{v}}$ *etc.*) have been applied to both of the detection Equations (4) and (5) under the premise that there is a time difference, it is impossible to guarantee the normal gait detection when an extremely high threshold is set. Therefore it is necessary to choose an appropriate threshold parameter to achieve the expected precision during the detection of pedestrian gaits.

**Figure 4:**
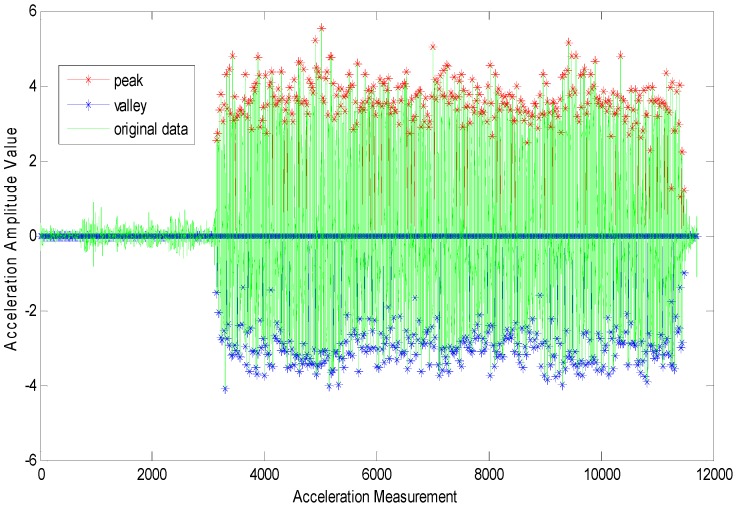
Gait detection based on acceleration data for walking state.

**Figure 5:**
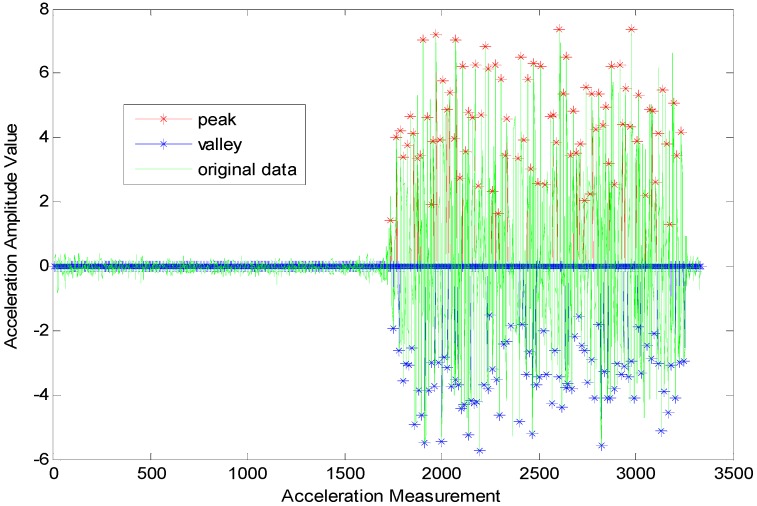
Gait detection based on acceleration data for running state.

On the other hand, the time-frequency parameter range in normal walking gaits is broader than that in a running state. The reason is that when the moving state is stable, it gives relatively stable acceleration data as shown in Figure 4. When the time threshold is set in a certain range (such as any number between 0.13 and 0.28 s), it will facilitate the accurate detection of the user’s step frequency. However, when people are running, the movement of their bodies (hands) will bring about lots of noise data among the acceleration data. As shown in Figure 5, the amplitudes vary very much in the numerical values. Then, it would be very hard to control gait detection when only an amplitude threshold parameter is applied. Hence, there are more requirements for time parameters, and the choice of an appropriate time threshold has become necessary for the accurate gait detection. For example, in run state, we are unable to guarantee an absolutely accurate detection unless the time threshold is set to be around 0.14 s.

As above, in order to unify the parameters that have been applied in the pedestrian gait detection model proposed in this paper, $\delta_{a}$, the amplitude threshold is set to be 1 m/s^2^. In terms of $\delta_{\Delta t}$, the time threshold for gaits, it is set to be 0.14 s, which is an appropriate numerical value that is applicable to the above four moving states. Moreover, in order to guarantee the accuracy of gait detection, the user must secure the stability of the device no matter at what speed he has walked in the positioning application process.

### 3.2. Step Length Estimation 

There is a positive correlation between the pedestrian’s step length and the acceleration data in terms of certain statistical values such as the following eigenvalues including the extremes, the variance and the periodicity of the acceleration, *etc.* Currently, the step length calculation model can be grouped roughly into a linear model and a non-linear model [30].

Regarding the linear step length model, Levi [31] proposed the concept of stride frequency detection and step length calculation in a pedestrian navigation system to establish the linear relationship between the step length and the stride frequency. After that, Ladetto proposed in [32] a linear relationship model with three parameters:

(6) SL=A+B×f+C×var+w

where *f* is the stride frequency, var is the acceleration variance of each step, w represents the Gaussian noise and *A–C* is the regression coefficient with all of these coefficients able to be obtained through learning and training. Moreover, in reference [33], a similar linear model with three parameters is proposed:

(7) $$SL=A+B\times p+C\times {\bar{s}}_{\max}$$

where *P* represents the gait cycle of every step and ${\bar{s}}_{\max}$ is the acceleration peak after the smoothing process. Same as above, *A–C* is the regression coefficient that can be obtained through learning and training. In reference [34], the step length calculation model has been analyzed according to the nonlinear concept. Such a nonlinear step length calculation formula with only two parameters involved is provided as below:

(8) $$SL=K\times \sqrt[{4}]{a_{\max}-a_{\min}}$$

where $a_{\max}(a_{\min})$ is the maximum (minimum) acceleration of every step, and *K* is the coefficient.

In this paper, an experiment on two linear models has been conducted, Equations (6) and (7), as well as a non-linear model, Equation (8), based on the acceleration statistics. Four persons of different heights and different sizes in different walking patterns are involved in the experiment for comparison and to research the feasibility and reliability of these three models in different walking patterns. Seven groups of data were chosen for walking/running at normal speed in the following modes including moving straight forward and turning a corner, *etc.* to accumulate all of the step values for the calculation of the user’s moving distances. Then, the calculated value was compared with the real distance, taking the distance difference as an index to test the reliability of the step length calculation model. The less distance difference proves that this model has better feasibility and reliability. After that, the least-squares method was utilized to obtain separately the following regression coefficients, $\Delta S_{LM}$ and *C_2_* = 0.068 for two linear models in addition to a coefficient *K* = 0.425 for the non-linear model. Then, the step length was calculated to obtain the following calculation results for the above three kinds of step length calculation models.

Where *S_LM_* and *S_NLM_* represent separately the displacement value of the pedestrian movement obtained after the computation is made based on the linear model and the non-linear model. $\Delta S_{LM}$ and $\Delta S_{NLM}$ represent separately the distance difference between the calculated distance and the real distance based on the linear model and the non-linear model. Table 2 shows that the distance error calculated separately based on these three models can be controlled within ±5 m. Actually, the average absolute distance differences obtained based on these three models are separately 1.555 m, 0.931 m and 2.032 m with the maximum absolute distance differences separately turning out to be 3.391 m, 2.299 m and 3.932 m. Their comparison reveals that the model established based on Equation (7) is the only model where the average absolute distance difference is lower than 1 m with all of the distance differences within ±3 m. Moreover, after the calculation, the variances of the absolute distance differences obtained based on these three models are separately 1.50, 0.61 and 2.04, proving that the second linear model is more stable. Hence, this paper takes the priority to adopt the Equation (7)-based model to obtain a real-time calculation of the step length.

**Table 2:** Normal gait step length calculation (unit: m).

	Real Distance						
	40.5	71.4	43.2	39.5	81.6	211.68	179
Linear model (Equation (6)) *S_LM1_*	38.608	73.766	43.348	40.421	81.526	213.771	182.391
Distance difference Δ*S_LM1_*	**−1.892**	**2.366**	**0.148**	**0.921**	**−0.074**	**2.091**	**3.391**
Linear model (Equation (7)) *S_LM2_*	41.411	71.372	44.799	38.894	82.381	209.381	178.707
Distance difference Δ*S_LM2_*	**0.911**	**−0.028**	**1.599**	**−0.606**	**0.781**	**−2.299**	**−0.293**
Non-linear model (Equation (8)) *S_NLM_*	38.427	73.065	46.227	39.895	81.520	215.612	175.950
Distance difference Δ*S_NLM_*	**−2.073**	**1.665**	**3.027**	**0.395**	**−0.080**	**3.932**	**−3.050**

### 3.3. Heading Estimation with Real-Time Compensation Based on Kalman Filter

When a person is walking with a cell phone held horizontally in hand, generally the azimuth of the cell phone can be considered as the heading angle of the person when he moves. The azimuth of the cell phone can be obtained through the computation made based on a gyroscope or a magnetometer. It is necessary to determine the initial direction before considering the adoption of a gyroscope to calculate the direction angle, which might suffer from a large error accumulation. However, the adoption of magnetometer is prone to the interference from the external signals, which would also give rise to big errors. Therefore, Krach *et al.* [34] has proposed introducing gyro data that is quite stable over a short time to compensate for the magnetometer, which is susceptible to interference for the purpose of calculating the temporal heading angle:

(9) $$h_{k}=(1-W)(h_{k-1}+w_{k}dt)+Wh_{mag,k}$$

where $h_{k-1}$ represents the directional data obtained at the previous moment, $w_{k}$ is the angular velocity in forward direction, $h_{mag,k}$ is the directional value currently obtained through the magnetometer and *W* represents the weight. Since gyro data can be represented with the angle variable of the direction, then it is feasible to apply *g*, its accumulated value, to judge the movement attributes of the user including turning a corner and moving forward.

**Figure 6:**
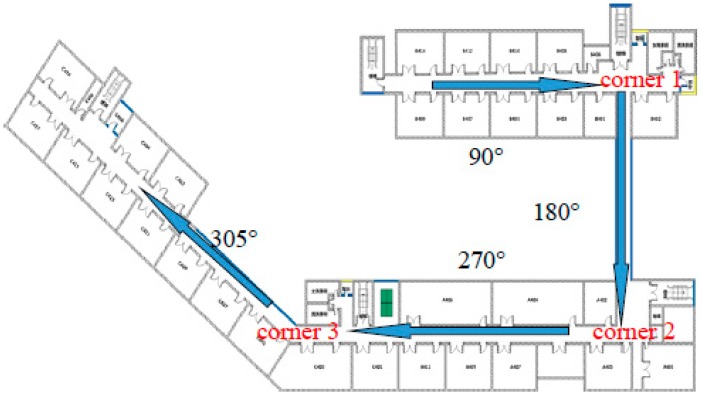
Walking trajectory.

**Figure 7:**
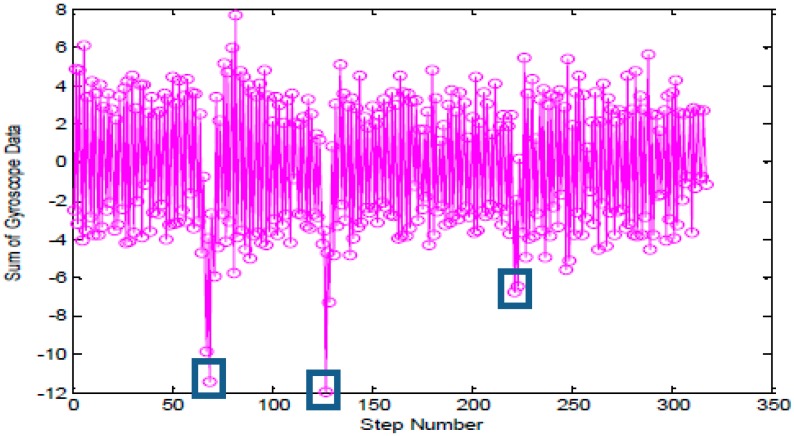
Analysis on the accumulated gyro value.

As shown in Figure 6, starting from the westernmost of Zone B, which is located on the fourth floor of the School of Environmental Science and Spatial Informatics (SESSI) building, walk along the corridor in the direction of arrow, where it reveals that the heading angle changes from 90° to 180°, then from 180° to 270° and finally to 305° according to the geographical orientation. As shown in Figure 7, three singular points representing separately the three turning processes when the users are walking can be seen in the accumulated gyro values. Hence, when *g* varies within the threshold range, basically it can be considered that the users are moving in a straight line, or else they are turning a corner.

Although improvements have been made as above, the deviation between the directional value, the output of the above equation and the real azimuth will become increasingly larger over time for the reason that the magnetic compass is prone to magnetic interference from the environment or from the platform. In [35], the various predictable errors are analyzed in detail including the following seven errors caused by the magnetic compass, such as the magnetic declination, the hard and soft-iron effects, the scaling factor and fixed bias, the installation misalignment and the movement of the pedestrian’s body with the reference of the other relevant documents. In order to simplify the calculation of the heading error model, a simplified expression is deduced in [36] to make an approximate determination of δ, which is the heading error:

(10) $$\delta =A+B\sin\xi^{\prime }+C\cos\xi^{\prime }+D\sin2\xi^{\prime }+E\cos2\xi^{\prime }+w$$

where $\xi^{\prime }$ is the azimuth value outputted from the digital compass and *A–E* refers to the five coefficients that should be corrected in the model.

This paper proposes a Kalman filter-based heading estimation method with real-time compensation according to the map geometry information. Through this method, it is necessary to re-train and correct the direction at each cornering to eliminate the accumulative errors in heading. According to this method, the coefficients (A,B,C,D,E) provided in Equation (10), which is also a heading error model, will be considered as the system state variables and the heading error will be considered as the system observation vector for the design of a Kalman filter to obtain the heading error in real time to compensate for the heading estimation.

To be specific, firstly perform filter smoothing on the data from an orientation sensor to get the original azimuth value, OriH by eliminating the error caused by the movement of body. Then, accumulate all of the gyroscopic values between peak and valley on the z-axis. After that, make a judgment on the moving state of the *i*th gait, which might be walking forward or turning a corner according to the value. In the case of walking forward, utilize the previous 10 steps to train the coefficients (A,B,C,D,E) in an error model according to the Kalman filter to get the heading error δ. In this way, all of the subsequent gait errors will be compensated in real time by δ, the error obtained according to the previous 10 steps. That is to say:

(11) heading(i)=OriH(i)+δ(i)

In the case of cornering, use the error mean for azimuth compensation:

(12) heading(i)=OriH(i)+mean(δ)

After every cornering, the coefficients (A,B,C,D,E) will be trained again in the error model according to the new map geometric direction value obtained when people are walking forward in a straight line after the cornering. In this way, the heading error arising in the direct movement after every cornering will be controlled perfectly to eliminate the accumulative heading error. In the 10 steps of training, the accumulative error at this moment is rather small, making it relevant to use directly the initial heading.

When X, the parameter of the heading error model is obtained through the Kalman filtering method, some of the key variables must be designed as below:

Define the state vector as the five calibration factors in Equation (10):

(13) X=[A,B,C,D,E]

The observation vector is defined as:

(14) Z=[FPH−H]

where *FPH* is the geometric azimuth that has been stored, and Matrix *H* represents OriH(i), the initial observation angle that has been stored.

The state vector coefficient is:

(15) $$KF\_H=\left[\begin{array}{l} \begin{array}{ccc} \begin{array}{ccc} 1\; & \sin(H(1))\; & \cos(H(1))\; \end{array} & \sin(2*H(1))\; & \cos(2*H(1)) \end{array}\\ \begin{array}{ccc} \begin{array}{cc} \begin{array}{cc} \vdots \; & \vdots \; \end{array} & \vdots \; \end{array} & \vdots \; & \vdots \end{array}\\ \begin{array}{ccc} \begin{array}{ccc} 1 & \sin(H(N_{H})) & \cos(H(N_{H})) \end{array} & \sin(2*H(N_{H})) & \cos(2*H(N_{H})) \end{array} \end{array}\right]$$

Assume that KF_P, the estimated variance matrix of state vectors is a diagonal matrix with the value of the entire diagonal elements set to be 1000, KF_Q, the dynamic noise matrix is a diagonal matrix with the value of the entire diagonal elements set to be 0.0001 and KF_R, the noise measurement matrix is set to be (5°)^2^.

**Table 3:** Comparative analysis for heading estimation error based on different methods.

	The Real Azimuth of 90°		The Real Azimuth of 180°		The Real Azimuth of 270°		The Real Azimuth of 305°	
	Initial Azimuth	Our Approach	Initial Azimuth	Our Approach	Initial Azimuth	Our Approach	Initial Azimuth	Our Approach
Mean difference	17.92	6.20	5.84	1.62	16.17	2.28	10.99	5.06
Maximum difference	28.63	15.81	12.09	4.06	22.72	17.52	20.54	18.18
Minimum difference	10.02	0.35	0.34	0.19	8.02	0.05	0.74	0.18

**Figure 8:**
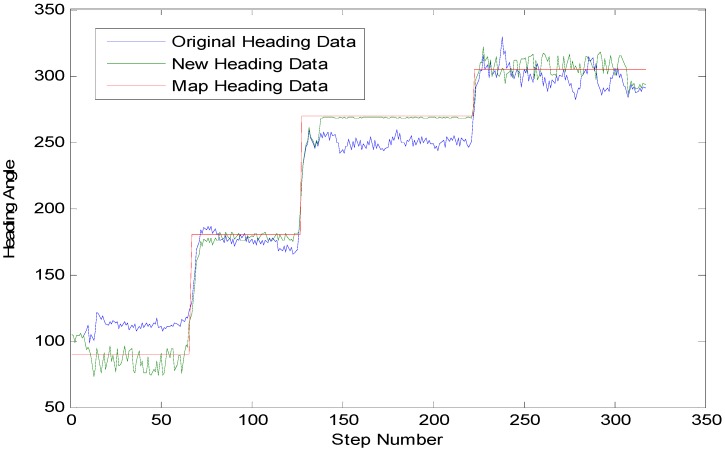
Test for heading estimation.

In Table 3, a comparison of the initial smoothed azimuth and the azimuth obtained after the real-time calculation of the compensation can be foubnd. The analysis on the pathway reveals that: On average, the heading errors with real-time compensation are separately 34.2%, 27.7%, 13.6% and 45.1% of the initial smoothed heading errors with the maximum angular difference, respectively, taking 53.8%, 33.7%, 77.8% and 90.3% of them and the minimum absolute error having been controlled basically within the range of 1°. Figure 8 shows very clearly that, through our heading estimation method, the heading has been improved significantly on the basis of the original azimuth in terms of the accuracy with the heading angle almost the same as the map heading data in red.

## 4. Positioning Integrated with Bluetooth and PDR

Set the relevant true position coordinates in the Bluetooth Beacon device according to its installation position with the signal emission frequency set to 1 Hz. Such an indoor positioning method based on the intensity of Bluetooth signal is able to provide the absolute position coordinates of the pedestrian without any error accumulation over time. On the other hand, since Bluetooth signal is prone to interference from the external environment, then only simple estimation of the position and area can be made through this method. If few Bluetooth Beacons are deployed, then only discrete prompts on the position can be provided. However, if too many Bluetooth Beacons are available, signal crosstalk will arise between the Beacons, leading to various instability phenomena, such as the skip of position or positioning failure, *etc.* In light of this, this paper has proposed and conducted an experiment on two fusion positioning methods based on Bluetooth and the PDR mechanism.

### 4.1. PDR Positioning Based on Map Matching and Bluetooth-Based Position Correction

This solution is completely subject to the map information regarding the direction calculation through the PDR method without the integration of Kalman filtering. Since only Bluetooth Beacons are required on some key waypoints, this method requires little layout work and maintenance, as well as little calculation work.

**Figure 9:**
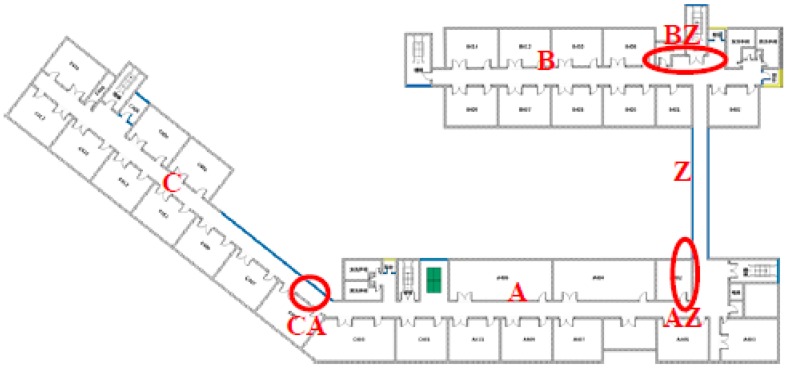
Cartographic zoning plan.

An indoor map for positioning purposes is stored in advance in the cell phone. As shown in Figure 9, the map is first segmented into several different zones. In our system, the map has been segmented into seven zones including Zone A, B, C, Z, BZ, AZ and CA in addition to the determination of the boundary coordinates in each zone. In our system, B, Z1, Z2, A1, A2 and C are defined as the coordinates of the central position at the entry of each linear zone.

In the map matching process, first segment the map into different regions, which consist of linear region and nonlinear region. The linear region is where users always walk in a straight line, such as in a corridor. However, in a non-linear region that is circled in red in the figure, users always turn a corner.

When people are walking, it is inevitable that their hands will swing to some extent, which will bring about certain error in heading estimation. In order to reduce such an error caused by hand swing, this paper adopts the mean of the directional values for the positions of the three acceleration extremes in a gait cycle, where generally there are two acceleration valleys and an acceleration peak. Then, the user’s movement attribute (turning a corner or walking straight on) is judged according to the accumulated variable value for the gyro angle during gait detection. If it indicates linear movement in a linear region, the direction angle for the region according to the magnetometer data and the map information should be adaptively identified. Or else, the mean directional value in a complete gait cycle as the heading angle of the step is taken.

Furthermore, in order to eliminate the heading errors accumulated in the process when the users are walking in a non-linear region, it is necessary to calibrate the users’ positions to eliminate error accumulation when the users are entering a linear region from a non-linear region. That is to say, when the users are moving from the non-linear region into the linear region, their position will be converted compulsively to the central position at the entry of the linear region.

### 4.2. Fusion Positioning Based on Adaptive Noise Extended Kalman Filter 

Through this solution, the dynamic model noise can be identified adaptively based on the moving information (walking forward or cornering). N,E represent separately the direction value of the acquired terminal coordinate in north and east, *s* is the step value obtained through gait detection for every step and θ is the angle value in horizontal moving direction. Assume that the position error, the step error and the heading error are the state variables of a filter system, then the state vector of the system can be expressed as:

(16) X=[dN,dE,ds,dθ]

Thus, the state equation for the EKF system is:

(17) $$\left\{ \begin{array}{l} dN_{k+1}=dN_{k}+\cos\theta_{k}\times ds_{k}-s_{k}\times \sin\theta_{k}\times d\theta_{k}+dw_{N}\\ dE_{k+1}=dE_{k}+\sin\theta_{k}\times ds_{k}+s_{k}\times \cos\theta_{k}\times d\theta_{k}+dw_{E}\\ ds_{k+1}=ds_{k}+dw_{s}\\ d\theta_{k+1}=d\theta_{k}+dw_{\theta } \end{array}\right.$$

Both the position coordinates and the dynamic displacement noise conform to the Gaussian distribution. That is to say, $w_{N}∼N(0,\delta_{N}^{2})$,$w_{E}∼N(0,\delta_{E}^{2})$, and $w_{s}∼N(0,\delta_{s}^{2})$. Considering a practical situation, the value of $w_{\theta }$ is rather big in the case of cornering and it is small in the case of direct movement regarding the azimuth. Then, it is applicable to determine adaptively the value of the dynamic noise that is related to azimuth through the recognition of the pedestrian’s moving state, which is in direct movement or cornering.

The state-transition matrix is:

(18) $$\Phi_{k}=\left[\begin{array}{l} \begin{array}{cccc} 1 & 0 & \cos\theta_{k} & -s_{k}\times \sin\theta_{k} \end{array}\\ \begin{array}{cccc} 0 & 1 & \sin\theta_{k} & \;s_{k}\times \cos\theta_{k} \end{array}\\ \begin{array}{cccc} 0 & 0 & 1 & 0 \end{array}\\ \begin{array}{cccc} 0 & 0 & 0 & 1 \end{array} \end{array}\right]$$

when the position coordinate of a Bluetooth system is obtained, take the position shift between the Bluetooth system and the PDR system as the observation value of the system to have the following observation equation:

(19) $$Z={\left[\Delta N,\Delta E\right]}^{T}={\left[N_{b,k}-N_{p,k},E_{b,k}-E_{p,k}\right]}^{T}$$

where (ΔN,ΔE) represents the position shift between two positioning systems at the moment of *k*, $\left(N_{b,k},E_{b,k}\right)$ is the positioning result obtained in a Bluetooth system at the moment of *k* and $\left(N_{p,k},E_{p,k}\right)$ is the position information obtained through the computation based on the dead-reckoning principle at the moment of *k*.

The observation matrix is provided as below:

(20) $$H_{k}=\left[\begin{array}{l} \begin{array}{cccc} 1 & 0 & 0 & 0 \end{array}\\ \begin{array}{cccc} 0 & 1 & 0 & 0 \end{array} \end{array}\right]$$

The dynamic noise matrix is:

(21) $$Q_{k}=\left[\begin{array}{l} \begin{array}{cccc} \delta_{N}^{2} & 0 & 0 & 0 \end{array}\\ \begin{array}{cccc} 0 & \delta_{E}^{2} & 0 & 0 \end{array}\\ \begin{array}{cccc} 0 & 0 & \delta_{s}^{2} & 0 \end{array}\\ \begin{array}{cccc} 0 & 0 & 0 & \delta_{\theta }^{2} \end{array} \end{array}\right]$$

Assume that $\delta_{N}^{2}=\delta_{E}^{2}=2$ and $\delta_{s}^{2}=1$. As to the value of $\delta_{\theta }^{2}$, it’s applicable to make a dynamic adaptive adjustment on it. When the pedestrian is moving forward in a straight line, $\delta_{\theta }^{2}={\left(2{}^{\circ}\right)}^{2}$ However, when the pedestrian is cornering, $\delta_{\theta }^{2}={\left(15{}^{\circ}\right)}^{2}$.

The noise measurement matrix is:

(22) $$R=\left[\begin{array}{l} \begin{array}{cccc} R_{w} & 0 & 0 & 0 \end{array}\\ \begin{array}{cccc} 0 & R_{w} & 0 & 0 \end{array} \end{array}\right]$$

Through the analysis of the statistical characteristics of the positioning errors in the Bluetooth system and the PDR system, an empirical value will be obtained, which is $R_{w}=10^{2}$.

In the case that the Bluetooth position has not been updated, take the difference between the coordinates predicted by the system and the coordinates observed through the PDR method as the observation variables to make a recursive correction on the calculation of the position through PDR with all of the others remaining unchanged.

(23) $$Z={\left[\Delta N,\Delta E\right]}^{T}={\left[N_{k+1}-N_{p,k+1},E_{k+1}-E_{p,k+1}\right]}^{T}$$

where the coordinates predicted by the system can be updated through the following approach

(24) $$\begin{array}{l} N_{k+1}=N_{p,k}+dN+(s_{k}+ds_{k})\times \cos(\theta_{k}+d\theta_{k})\\ E_{k+1}=E_{p,k}+dE+(s_{k}+ds_{k})\times \sin(\theta_{k}+d\theta_{k}) \end{array}$$

According to the position error obtained through the filtering, Equation (24) is able to update the pedestrian’s current position, which is also the final position at the moment of *k + 1* obtained after the computation made in the fusion model.

## 5. Experimental Section

A test field has been constructed on the fourth floor of the School of Environmental Science and Spatial Informatics (SESSI) building in China University of Mining and Technology (CUMT) to build a Bluetooth-based positioning system with the Samsung Galaxy Note3 (SM-N9002) selected as the mobile testing device. The Bluetooth Beacon will be deployed along the corridor in the test field. During the positioning, the frequency for Beacon positioning is set to be 1 Hz and the data sampling frequency of the inertial sensor is 50 Hz. Every time when the positioning system receives new Bluetooth coordinates, position correction of the positioning results obtained through the Bluetooth and through the PDR method will be made in the appointed fusion model. In the experiment, the pedestrian will walk along the corridor from the westernmost of Zone B on the fourth floor to the westernmost of Zone C across Zone A at normal speed (1.5 m/s~1.8 m/s). In this process, the pedestrian has to walk in total 316 steps with the device held in hand horizontally. Then, through the computation on the position errors and the error distribution according to different positioning solutions, this paper makes an evaluation of the performance and reliability of these solutions.

This paper puts forward four solutions to conduct the positioning experiments involved with three heading estimation methods: The first method has been used to conduct a smoothing process on the data from the orientation sensor in a gait cycle with the adoption of the mean value as the azimuth value of this step. The second method is the geographical azimuth-matching method and the third method, which is also a heading estimation method with real-time compensation, has been proposed in this paper according to Kalman filter with the details provided as below:

Solution I: This solution is named original heading PDR, flagged as OHPDR, which is a PDR-based positioning method based on the original smoothed azimuth.

Solution II: This solution is named adaptive heading PDR, flagged as AHPDR, which is a PDR-based positioning method with real-time compensation for the azimuth based on the filtering.

Solution III: This solution is named Bluetooth-based PDR, flagged as BEPDR, which is a PDR-based positioning method based on map matching with Bluetooth-based position correction.

Solution IV: This solution is named EKF-based PDR, flagged as EKFPDR, which is a fused positioning method integrated with Bluetooth and AHPDR based on the adaptive noise EKF.

**Figure 10:**
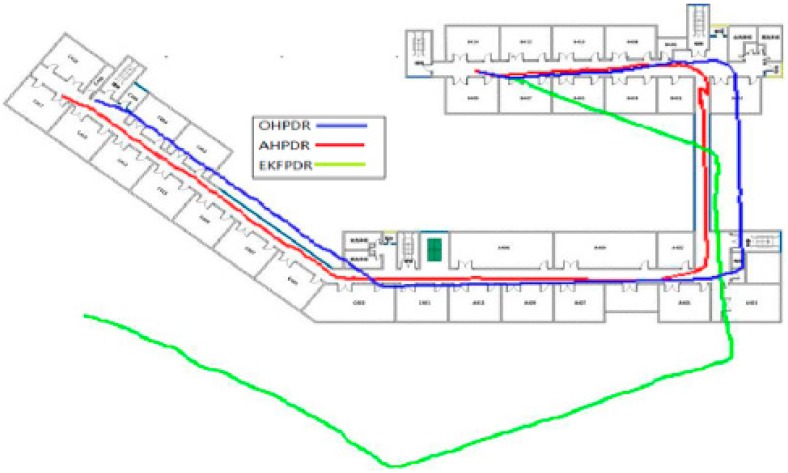
Positioning trajectory analysis for different solutions associated with PDR.

Solution I is a dead-reckoning approach based on the original smoothed azimuth with the positioning error turning out to be increasingly bigger over time. Actually, even in an extremely short time of around 5 min, the error accumulated from the initial 2 m to 44.85 m as indicated in Figure 10, where the trajectory is shown in blue.

Solution II provides an online solution for the parameters of the heading error model to realize a real-time compensation and correction of the pedestrian’s heading information by calculating the heading error based on the initial 10 mean azimuth values and the map directional information. On average, the PDR method based on such an azimuth is able to control perfectly the positioning error within 5 m with the maximum error at only 6.19 m, which proves that such a PDR-based positioning solution with heading correction is able to eliminate the problem of error accumulation as shown in Figure 10, where the trajectory is shown in red.

**Figure 11:**
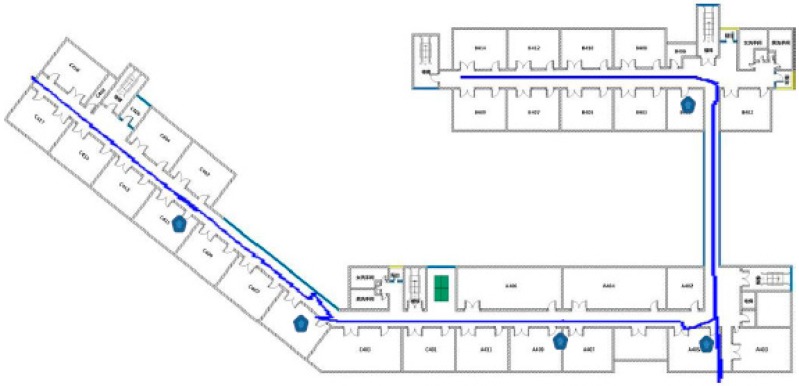
PDR-based positioning method based on map matching with Bluetooth-based position correction.

In Solution III, the heading trajectory is determined directly based on the map matching method. That is to say, the map geometric direction values (such as 90°, 180°, 270° and 305°) will be adopted directly in a linear region. Although this method is able to express perfectly the geometric shape of the corridor, it requires high-precision gait detection and step length calculation, making it hard to adopt the PDR algorithm, which is affected negatively by speed and other personal characteristics. Moreover, since there are errors in gait detection and step length calculation, it would be prone to deviate from the real building structure. For example, as shown in Figure 11, “cross-wall” phenomenon can be found in the walking trajectory at the second corner. In order to make a correction to the trajectory, five Bluetooth Beacons are deployed at some key inflection points. The advantage is that it will correct the deviated trajectory. However, such a compulsory correction will lead to the partial overlapping of the trajectory. That is to say, “go-and-back phenomenon” can be observed on the trajectory to make the whole trajectory look unnatural. However, through this method, the mean error can be controlled within about 4 m.

In Solution IV, 20 Beacons are deployed every 10 m throughout the whole corridor. Then, a fusion calculation is made based on the positioning result through these 20 Bluetooth systems according to the adaptive noise EKF adopted in AHPDR as shown in Figure 10, where the trajectory is shown in green. This solution has worked very well not only in solving the problem of serious error accumulation that can be found through the traditional PDR method, but also in the significant improvement of the positioning precision. Compared with the previous three solutions, this solution has reduced separately the minimum error by 87.5%, 87.5% and 26.1%, reducing the mean error respectively by 90.2%, 58.5% and 45.1%. Also, compared with Solution I and Solution III, the maximum error has been reduced by 88.4% and 58.3%. However, due to the positioning error in a Bluetooth-based system, the maximum error in Solution IV is equivalent to that in AHPDR with a difference of only 1.7 m.

**Figure 12:**
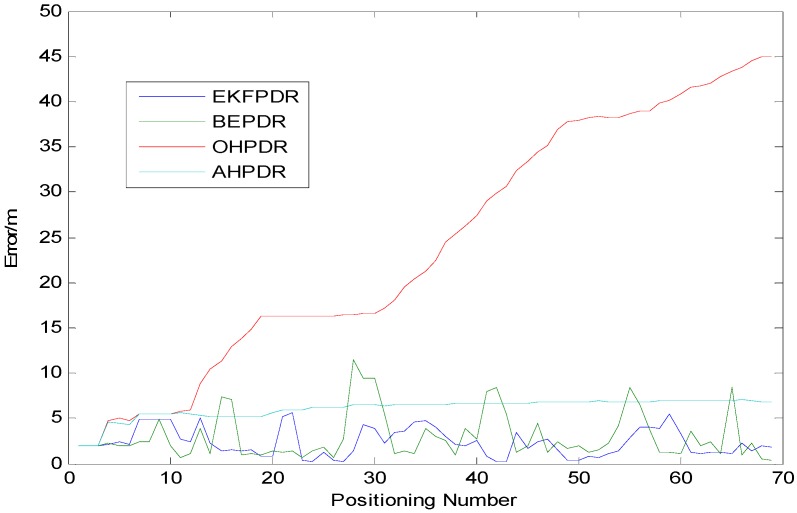
Positioning trajectory analysis for different solutions associated with PDR.

Table 4 and Figure 12 show the positioning errors arising in the above four solutions:

**Table 4:** Positioning error analysis for different solutions associated with PDR.

	OHPDR	AHPDR	BEPDR	EKFPDR
Min. error/m	2.00	2.00	0.34	0.25
Mean error/m	22.67	5.42	4.12	2.26
Max. error/m	44.85	6.79	12.21	5.09

**Figure 13:**
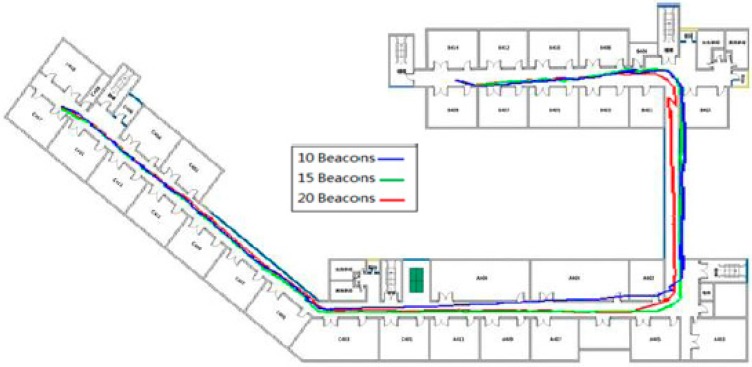
Positioning trajectory analysis for different numbers of Beacons.

Moreover, in order to verify the relationship between the number of Bluetooth Beacons that have been deployed and the positioning result, we deployed separately 20, 15 and 10 Beacons along the corridor with the positioning results shown in Table 5 and Figure 13 after the integration of the PDR algorithm. According to Table 5 and Figure 13, the more Beacons that are deployed, the better the filtering effect is from the perspective of the overall positioning result. Also, from the perspective of mean error, the fusion result based on 20 Beacons is better than that based on 15 and 10 Beacons with the error reduced separately by 43.8% and 28.9%. However, it is not true that the more Beacons that are deployed, the better the effect is due to the crosstalk between the Beacon signals. In an ideal situation, the separation distance of the Beacons that have been deployed should be at least twice the radius of the specified detection area.

**Table 5:** Positioning error analysis for different numbers of Beacons.

Beacons	10	15	20
Min. error/m	0.41	0.36	0.25
Mean error/m	3.97	3.15	2.26
Max. error/m	5.95	5.42	5.09

As mentioned above, in order to eliminate the heading error accumulation, it is feasible to make a filtering correction through the constraints on map information or based on the PDR result. Meanwhile, on the basis of this, the integration of the Bluetooth-based positioning system and the PDR method according to EKF is able to achieve a 2 m positioning precision. However, this method will not work well in the case that the walking route is irregular or in some irregular and complex fields, where only fusion positioning through OHPDR and the Bluetooth-based positioning system can be adopted. Moreover, when too many Bluetooth Beacons are deployed, it would be a big burden to replace all of the batteries. Therefore in the case that high-precision positioning is not required, Solution III would be a compromise with only some key inflection points deployed with the Bluetooth.

## 6. Conclusions

Based on the particular advantages of these two positioning systems using the Bluetooth Beacon and the PDR method, this paper proposes two fused positioning solutions following an improvement analysis of the corresponding gait detection, step length calculation and heading calculation through the PDR algorithm. One is a PDR-based positioning method based on map matching and Bluetooth-based position correction and the other method is based on an adaptive system and dynamic noise filtering to make a fusion calculation according to the pedestrian’s moving state (walking forward or cornering). The experimental results prove that when indoor positioning is conducted according to the PDR technology, the correction to the pedestrian’s azimuth is able to efficiently inhibit the error accumulation using the PDR method. Meanwhile, the integrated application of different technologies in both the Bluetooth Beacon system and the PDR-based positioning system is able to effectively solve the “go-and-back” problem found in the positioning using the Bluetooth Beacons and the problem of positioning error accumulation using the PDR method to improve both the reliability and robustness of the indoor positioning.
